# Lipidomics and anti-trypanosomatid chemotherapy

**DOI:** 10.1186/s40169-017-0160-7

**Published:** 2017-08-01

**Authors:** Michael Biagiotti, Sedelia Dominguez, Nader Yamout, Rachel Zufferey

**Affiliations:** St John’s University, 8000 Utopia Parkway, Queens, NY 11439 USA

**Keywords:** Lipids, Mass spectrometry, Kinetoplastid parasite, Drug target, *Trypanosoma brucei*, *Trypanosoma cruzi*, *Leishmania*

## Abstract

**Background:**

Trypanosomatids such as *Leishmania, Trypanosoma brucei* and *Trypanosoma cruzi* belong to the order Kinetoplastida and are the source of many significant human and animal diseases. Current treatment is unsatisfactory and is compromised by the rising appearance of drug resistant parasites. Novel and more effective chemotherapeutics are urgently needed to treat and prevent these devastating diseases, which relies on the identification of essential, parasite specific targets that are absent in the host. Lipids constitute essential components of the cell and carry out multiple critical functions from building blocks of biological membranes to regulatory roles in signal transduction, organellar biogenesis, energy storage, and virulence. The recent technological advances of lipidomics has facilitated the broadening of our knowledge in the field of cellular lipid content, structure, functions, and metabolic pathways.

**Main body:**

This review highlights the application of lipidomics (i) in the characterization of the lipidome of kinetoplastid parasites or of their subcellular structure(s), (ii) in the identification of unique lipid species or metabolic pathways that can be targeted for novel drug therapies, (iii) as an analytic tool to gain a deeper insight into the roles of specific enzymes in lipid metabolism using genetically modified microorganisms, and (iv) in deciphering the mechanism of action of anti-microbial drugs on lipid metabolism. Lastly, an outlook stating where the field is evolving is presented.

**Conclusion:**

Lipidomics has contributed to the expanding knowledge related to lipid metabolism, mechanism of drug action and resistance, and pathogen–host interaction of trypanosomatids, which provides a solid basis for the development of better anti-parasitic pharmaceuticals.

## Introduction

Trypanosomatids are unicellular protozoan parasites that belong to the order of Kinetoplastida. They are characterized by the presence of an unusually dense disc-like mitochondrial DNA structure, the kinetoplast, which is located at the basis of flagellum’s attachment the site to the cell body. Trypanosomatids are the ethiologic agents of three major human diseases, sleeping sickness, Chagas disease, and leishmaniasis, which are caused by *Trypanosoma brucei*, *Trypanosoma cruzi*, and by various species of *Leishmania*, respectively. Altogether, they affect about 37 million people every year, mainly in the tropical and subtropical area of the world [1]. All three trypanosomatids undergo a complex life cycle alternating between an insect vector and a vertebrate host. *T. cruzi* is transmitted via the feces of the triatomine bug (kissing bug) where it exists as epimastigotes and differentiates into metacyclic trypomastigotes before transmission into the vertebrate host. In the latter, *T. cruzi* infects various cell types where trypomastigotes differentiate into intracellular amastigotes. Similarly, *Leishmania* parasites develop as flagellated promastigotes within the digestive tract of the female sandfly, which bites a vertebrate host to transmit the parasite while taking a blood meal. Promastigotes are phagocytized mainly by macrophages of the vertebrate host, where they differentiate into non-motile amastigotes. *T. brucei* develops mainly as procyclic forms in the tsetse fly insect vector, and multiplies extracellularly in the bloodstream of a mammal as bloodstream forms. Treatment of these parasitic diseases remains poorly effective and is complicated by the growing appearance of drug resistant parasites. No effective vaccines exist yet against these pathogens and thus, the need to develop novel pharmacological agents is highly desired.

Parasites’ lipids have attracted much attention in the last two decades for many obvious reasons that support the idea that lipid metabolism can be targeted for drug design. Lipid production is a necessary prerequisite for the rapid multiplication of parasites and for the establishment of infection. Further, lipids fulfill numerous essential functions in the parasite’s biology. Lipids, as part of biological membranes, provide a platform of interaction between the parasite and the host as parasites penetrate or are taken up by host cells. Parasites are typically unable to produce their whole assortment of lipids and thus, they need to scavenge host’s lipids or host’s lipid precursors in order to meet their cellular demand [2–4]. Several lipid-based macromolecules function as virulence factors, such as lipophosphoglycan of *Leishmania* and glycosylphosphatidylinositol (GPI)-anchored proteins, which contribute to the establishment of infection and modulation or evasion of the host’s immune system (reviewed in [5]). Lastly, lipid-based drugs have been tested in pre-clinical trials for the treatment of parasitic diseases [6].

Lipidomics is the newest “omics” sub-discipline of metabolomics that has come to exists since 2003 only, and aims to quantify and identify all lipids (lipidome) of a cell or of a tissue. It provides snapshots of the lipid composition of a cell/tissue under a specific or different conditions and allows profiles’ comparison. In addition, it enhances the knowledge of lipid function and regulation at the level of individual species, and of specific molecules on lipid metabolism. Lastly, lipidomics focuses on elucidating novel structures of lipids. This review summarizes the applications of lipidomics in the advancement of understanding the biology of *T. brucei*, *T. cruzi*, and *Leishmania* parasites in terms of lipid content and lipid metabolic pathways. The use of lipidomics in unravelling the mechanisms of action of anti-parasitic drugs and of drug resistance is also discussed. Lastly, an outlook describes future directions where this field is evolving.

## Structures and functions of lipids

Lipids are essential macromolecules that are found in high abundance in all organisms. They were originally defined as hydrophobic molecules and fulfill various functions such as energy storage, physical barriers in form of biological membranes, and signaling as regulatory molecules. It has become increasingly evident that lipids, through their complexities, have arisen as vital factors controlling numerous cellular processes. Impairment of lipid metabolism is the cause of many human diseases such as insulin-resistant diabetes, cancer, Alzheimer’s disease, atherosclerosis, obesity, steatohepatitis, sterility, Barth’s syndrome, heart failure, brain function, hearing loss, immune deficiency, and liver disease (reviewed in [7–9]).

**Fig. 1:**
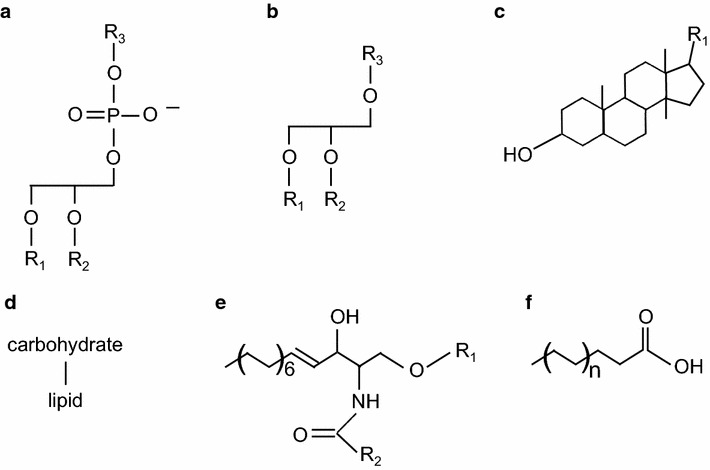
Schematic representation of the six different classes of lipids. **a** Glycerophospholipid. *R*_*1*_ represents a fatty acyl or alkyl groups. *R*_*2*_ and *R*_*3*_ depict a fatty acyl group and a polar group, respectively. **b** Glycerolipid. *R*_*1*_ represents a fatty acid while *R*_*2*_ and *R*_*3*_ can be a fatty acid or a hydroxyl group. **c** Steroid based lipid. **d** Glycolipid. The lipid anchor can be a glycerophospholipid or a sphingolipid. **e** Sphingolipid. *R*_*1*_ and *R*_*2*_ represent a polar group and a fatty acyl group, respectively. **f** Free fatty acid

Lipids are made of a small number of building blocks, however, they are immensely diverse and relatively complex. Their synthesis originates from the condensation and reduction of only two precursor molecules that include a ketoacyl or isoprene unit. They can be classified into six main lipid categories: glycerophospholipids [phosphatidylcholine (PC), phosphatidylethanolamine (PE), phosphatidylinositol (PI), phosphatidylserine (PS), phosphatidic acid (PA), cardiolipin (CL), phosphatidylglycerol (PG)], glycerolipids (triacylglycerol, diacylglycerol, monoacylglycerol), glycolipids, sterols, sphingolipids (SP), and free fatty acids (Fig. 1). Each individual lipid species are essential components of a living organism and therefore, highlighting the importance of the identification and quantification of the lipidome of an organism is highly desired.

## Application of mass spectrometry in lipidomics

Lipidomics rests primarily on the development of the mass spectrometry (MS) technology. MS is usually coupled to a chromatographic method such as gas chromatography (GC), which is typically used to separate smaller lipids such as free fatty acids or sterols, or liquid chromatography (LC), which is commonly applied to fractionate low abundance lipid species. Occasionally, thin layer chromatography is carried out to separate different types of lipid species before MS analysis. The analysis of the whole lipid sample without prior chromatographic fractionation is referred to as shotgun lipidomics or direct-infusion lipidomics. The current, most common ionization techniques are electrospray ionization (ESI; soft ionization), nanoESI, and MALDI-TOF (matrix assisted laser desorption/ionization coupled to time-of-flight). Further structural elucidation is routinely achieved by MS–MS, where after analyte’s collision, characteristic fragments (products ion scan) such as polar head groups (or their parts thereof), fatty acids, long chain bases or other fragments are captured. Moreover, neutral losses can be generated. Addition of small cations or organic acids to the matrix allows alkaline adducts to be analyzed in the positive mode while supplementation of small anions to the matrix permits anionic adducts or deprotonated lipid fragments to be examined in the negative mode. Typical analyzers are time of flight (TOF), quadrupole, the ion trap, and Fourier-transform ion cyclotron resonance (FTICR-MS) analyzer. To distinguish isobars, high resolution MS can be achieved using orbitrap or Fourier transform-ion cyclotron resonance instruments. Lipids can be analyzed individually by the so called targeted lipidomics approach. In contrast, the untargeted or global lipidomics permits the identification of the whole lipidome of a cell or a tissue.

## Investigating the lipidome of trypanosomatids

### Lipidome of *L. donovani* insect and vertebrate cell stages

*Leishmania donovani* is responsible for the lethal, visceral form of leishmaniasis in the Old World. To gain an insight into cell stage-specific changes in parasite’s lipid metabolism, Bouazizi-Ben et al. analyzed the lipidome of both the promastigotes and amastigotes of *L. donovani* [10]. Free fatty acids and sterols were analyzed by GC–MS while PL were examined by HPLC–MS. The salient differences in lipid content between both cell stages is the cholesterol/PL ratio, which reflects the twofold increase of cholesterol quantities associated with a modest decrease in PL content in the vertebrate form. Notably, *Leishmania* does not produce cholesterol and thus, it exclusively scavenges it from the host (reviewed in [11]). Furthermore, the free fatty acid pool increased twofold in amastigotes likely resulting from the hydrolysis of TAG and PL. Major PL species in *L. donovani* and *L. infantum* promastigotes are PC, PE, followed by PI and very low levels of PS, CL, PG, and PA. Amastigotes harbored elevated amounts of PS and sphingomyelin (SM) but lower quantities of PE and PI compared to promastigotes. The latter contained higher amounts of unsaturated fatty acids compared to saturated ones with C18 (saturated or unsaturated) being the dominant fatty acid in PL, TAG and free fatty acids. Promastigotes synthesized proportionally more n-6 than n-3 polyunsaturated fatty acids than amastigotes. Amastigotes harbored more saturated and monounsaturated fatty acids in its glycerolipids but decreased amounts of n-6 unsaturated fatty acids, regardless of the lipid class, which is similar to the fatty acid content of macrophages, indicating that remodeling of lipid depends on the activity of fatty acids desaturases present in macrophages.

Sterol profiling of *L. infantum* procyclic (replicating; non virulent) and metacyclic (non-dividing; virulent) promastigotes by GC–MS revealed that it contains cholesterol, two isomers of ergosterol, ergosta-7,22-dien-3β-ol, and stigmasta-7,24(28)-dien-3β-ol, and the sterols ergostatetraenol, an additional isomer of ergosta-7,22-dien-3β-ol, zymosterol, and lanosterol [4]. Ergosterols were more abundant in multiplying promastigotes than in metacyclic parasites while the opposite situation applies to cholesterol, ergosta-7,22-dien—I and II and stigmasta-7,22-dien. The authors hypothesized that dynamic changes in sterol composition during parasite development promotes the differentiation of procyclic to metacyclic promastigotes (metacyclogenesis).

### Identification of new lipid species in *L. infantum*

*Leishmania infantum* is responsible for visceral leishmaniasis in the Mediterranean region. A targeted, high resolution lipidomics approach was applied to fully characterize rare, unusual lipids species in *L. infantum*. This technology allowed the identification of the unusual dimethyl–PE in this strain of parasite, and the presence of rare cyclopropane fatty acyl (CFA) chain containing PE and CFA containing plasmalogen PEs [12]. CFAs are typically absent in mammalian cells but are widely present in bacteria such as *Escherichia coli* and *Mycobacterium tuberculosis* (reviewed in [13]). More importantly, CFA are only present in *L. infantum*, *L. brasiliensis*, and *L. mexicana* and thus, can be used as a diagnostic criteria [14]. The function of CFA in *Leishmania* is however unclear.

### Differences in *T. brucei* procyclic and bloodstream forms’ lipidomes

Although the lipid content of *T. brucei* was characterized previously [15], a global lipidomics approach involving the shotgun method provided a deeper and more precise “map” of the lipidome of both procyclic and bloodstream forms of the parasite [16]. The salient differences between the procyclic and bloodstream forms are the presence of inositolphosphoceramide (IPC), diacyl and ether PI species containing shorter fatty acids in procyclic forms, while these lipids were absent in the bloodstream form [16, 17]. In contrast, SM was found in both cell stages but they were more abundant in bloodstream forms. While the levels of glycerophospholipids were not drastically altered, difference in the nature of the fatty acids was observed; procyclic forms bear more unsaturated fatty acids in their PL compared to bloodstream forms. Diacyl–PE are enriched in bloodstream forms compared to procyclic trypanosomes but PG and PS levels were unchanged in both forms of the parasite.

### The flagellar membrane of *T. brucei* contains low amounts of PC and PI

Trypanosomatids possess a single flagellum that is anchored to the cell body via the flagellar pocket, an invagination of the plasma membrane, and is connected to the cell body for most of its length. Due to its importance in movement, parasite morphogenesis, and pathogenicity, the flagellar structure and composition attracted interest among parasitologists (reviewed in [18, 19]). The flagellar membrane is an extension of the flagellar pocket membrane and lays adjacent to the plasma membrane. Reverse phase liquid chromatography MS–MS (RPLC–MS–MS) analysis of the lipidome of the flagellar membrane fraction revealed that it contained higher amounts of PE, PS, ceramide, IPC, SM, and ether glycerolipids [20]. In contrast, the flagellar membrane barely contained any PC and PI compared to the whole cell content.

### Glycosomal PC content and the role of glycosomal division proteins GIM5A/B in lipid metabolism

Glycosomes are evolutionary peroxisomes-related organelles [21]. They harbor among others the first seven glycolytic enzymes, the purine salvage pathways, and several ether lipid biosynthetic enzymes (reviewed in [22, 23]). Glycosomes are organelles unique to trypanosomatid parasites and thus, they have become the focus of many investigations, including “omics” approaches. One puzzling topic of inquiry is how molecules are transported across the peroxisomal membrane. In order to answer this question, glycosomal PC species of highly purified glycosomes were analyzed by targeted lipidomics using the shotgun technique [24]. No major differences were detected between the PC composition of glycosomes and whole cell extracts isolated from both procyclic and bloodstream forms of *T. brucei*. The authors concluded that the permeability of the glycosomal membrane is similar to that of other membranes of the cell, including the impermeability towards polar molecules.

As glycosomes harbor ether lipid biosynthetic enzymes (reviewed in [22, 23]), the role of glycosomal division proteins GIM5A and GIM5B in parasite physiology and lipid metabolism were investigated [25]. Deletion of the *GIM5A* gene did not affect the growth of bloodstream forms, but depletion of *GIM5B* in a *Δgim5a* null background was lethal. *GIM5A* was dispensable for procyclic trypanosomes survival even when *GIM5B* was down-regulated. *GIM5A/GIM5B* depleted cells possessed fewer glycosomes than the wild type and were hypersensitive to osmotic stress. The mutant cells’ PC and PE species, which account for over 70% of all phospholipids found in *T. brucei* [15] were analyzed by HPLC–MS. *GIM5A* deletion alone (*Δgim5a*) induced 40–70% reduction in ether PE and ether PC contents [25]. However, the mechanism(s) by which GIM5A affects ether lipid biosynthesis is unknown.

## Structure determination and biosynthesis of GPI-anchors

GPI-anchors are glycolipids that tether proteins to the biological membrane in eukaryotic cells. Many GPI-anchored proteins in trypanosomatids are involved in virulence or host immune evasion [5]. A targeted lipidomics approach involving the direct infusion of the sample allowed the elucidation of the structure of the GPI-anchor in *T. congolense*, which causes nagana, a disease of cattle [26]. These studies demonstrated that GPI-anchors of procyclic forms are a heterogenous family of PI species, carrying one acyl or two acyl linked to the glycerol moiety, or three acyl groups where two are attached to glycerol and the third one to inositol. Some of these species are myristoylated at the *sn*-2 position. In term of biosynthesis, GPI-anchored glycoconjugates initially receive tri-acylated GPI-precursors, which are subsequently de-acylated either at the glycerol backbone or on the inositol ring. However, the GPI-anchor structure of *T. congolense* procyclic forms’ GARP (glutamic acid and alanine rich protein) was determined by MS and was found to be homogenous, consisting of an acylated inositol and a diacyl–PI, where the *sn*-2 position of the glycerol backbone is occupied either by a myristic or oleic acid [27]. It seems that in this strain of Trypanosomes, GPI-anchor structures are proteins specific.

Targeted lipidomics using the shotgun method was attempted to confirm the structure of the glycoforms of variant surface glycoprotein (VSG), which covers the plasma membrane of *T. brucei* bloodstream trypanosomes and is involved in host’s immune evasion [28–31]. However, this technique failed to provide structural information about the branching pattern of and types of covalent bonds within the various GPI glycoforms of VSG.

The cell surface of *T. cruzi* is covered with mucin-like sialic acid acceptors that are GPI-anchored into the plasma membrane (reviewed in [32]). These molecules are essential for host cell invasion by metacyclic trypomastigotes [33, 34]. Targeted MS analysis of these mucins from non-infective epimastigotes and metacyclic trypomastigotes established that the lipid moiety of the GPI-anchor of epimastigotes consists of a 1-*O*-hexadecyl-2-*O*-hexadecanoyl-PI while metacyclic trypomastigotes harbor IPC with C24:0 and C16:0 fatty acids as lipid anchor instead [35]. These cell stage-dependent differences in the structure of lipid anchors may account for the inability of epimastigotes to infect mammalian cells.

Global lipidomics applying the shotgun procedure was instrumental in identifying the defect in GPI-anchor biosynthesis in mutant cell lines lacking the *TbGPI12* gene in procyclic trypanosomes [17]. Such mutant strain accumulated GlcNAc-PI species with C18 long fatty acids, demonstrating that the *Tb*GPI12 enzyme catalyzes the second step in GPI-anchor biosynthesis by acting as a Glc-NAc-PI de-*N*-acetylase [17].

## Variations in sphingolipid metabolism in trypanosomatids

### Sphingolipid metabolism in *Leishmania* is dispensable for viability but is essential for ethanolamine production

Similar to yeast, *Leishmania* promastigotes and amastigotes synthesize IPC [36–40]. These sphingolipid species are absent in the vertebrate host and thus, the importance of these lipids in parasite’s biology attracted significant interest. Rightfully so, IPCs were found to be important for vesicular trafficking, differentiation from avirulent to virulent promastigotes during the stationary phase of growth (metacyclogenesis), acidocalcisome biogenesis, and in in vivo virulence [38–41]. The structure of *Leishmania* IPCs were thus extensively analyzed by targeted lipidomics. It was found that the predominant ceramide carries the 16:1 base and the lesser component bears the 16:0 base but both contain the *N*-stearoyl group [39, 42, 43].

Based on global MS analysis of the lipidome of the promastigote mutants sphingosine 1-phosphate lyase *spl* ^−*/*−^ and serine palmitoyltransferase *spt2* ^−/−^, both strains produced IPC and ceramide but harbored decreased levels of PE and PC [38, 41]. These analyses also revealed that stationary parasites produced larger amounts of plasmalogen PE, which are likely important for metacyclogenesis [41]. Analysis of the lipidome of *spt2* ^−/−^ mutant amastigotes established that they still synthesized IPC, very likely by head group remodeling of complex sphingolipids [38], a mechanism that is absent in humans. In contrast to other eukaryotes, sphingolipids are dispensable to *Leishmania* viability but catabolism of SL has instead evolved to be the major route for ethanolamine biosynthesis, and thus, *Leishmania* is ethanolamine prototroph [41, 44]. Mutant lacking the *ISCL* (inositol phosphosphingolipid phospholipase C-Like) gene produced IPC, plasmenylethanolamine and ceramide, as per ESI–MS analysis of its lipidome, but failed to form lesion in mice, suggesting that the parasite relies on degradation of host SM or SL for infectivity as the parasite lacks the capability to produce SM [3].

## Differential expression of sphingolipids in *T. brucei*

### *T. brucei* synthesizes all three types of sphingolipids but in a cell stage dependent fashion

*Trypanosoma brucei* is unique among eukaryotes in that it synthesizes all three types of sphingolipids, IPC, SM, and ethanolaminephosphoceramide (EPC; [16, 45]). However, they are differentially expressed; procyclic trypanosomes contain IPC and SM, while bloodstream stage parasites produce EPC in addition to SM but no detectable IPC [45]. Expression of *Tb*SLS4 in *Leishmania* followed by analysis of the lipidome of the transgenic line by the shotgun approach resulted in production of both SM and EPC, demonstrating that *Tb*SLS4 exhibits bi-functional synthase activity. RNAi silencing of *TbSLS1-4* in bloodstream trypanosomes led to abrupt growth arrest followed by cell death and accumulation of ceramide, suggesting that this important signaling molecule mediated a toxic downstream effect [45].

LC–MS analysis of the products of the putative bifunctional sphingolipid D4-desaturase/C4-hydroxylases of *T. brucei*, *L. major*, and *T. cruzi* demonstrated that these enzymes are capable of desaturation or hydroxylation of sphingoid bases [46]. The detection of such structures in whole cell extracts of *T. cruzi* epimastigotes, *L. major* promastigotes, and *T. brucei* procyclic and bloodstream forms are consistent with the presence of such enzymes in these organisms.

## Delineation of glycerophospholipid biosynthetic pathways by lipidomics

### PE is made exclusively via the de novo pathway and is essential for the viability of *T. brucei* bloodstream forms

Labeling of cells with D4-ethanolamine followed by shotgun lipidomics lead to the incorporation of this precursor in PE species only and no labeling was found in PC, demonstrating the absence of cross talk between the PE and PC de novo pathways [16]. This situation is very unusual as mammalian cells and other parasites express PE methyltransferases that convert PE into PC while plants and *P. falciparum* possess a phosphoethanolamine methyltransferase [47–49]. The absence of PE and phosphoethanolamine methyltransferase genes in *T. brucei* genome is consistent with this biochemical result. Further, D_3_-serine incorporated only in PS and not in PE, demonstrating that PS decarboxylation does not occur in *T. brucei* and that PE is exclusively made via the Kennedy pathway [16, 50]. However, *T. brucei* possesses a PS decarboxylase, *Tb*PSD, but its function is not in the decarboxylation of PS [51].

The cytosolic ethanolaminephosphate cytidylyltransferase *Tb*ECT catalyzes the formation of CDP-ethanolamine from ethanolaminephosphate, in a CTP and magnesium dependent fashion [50]. In vivo metabolic labelling of *TbECT* depleted cells followed by analysis of total cellular phospholipids using the shotgun technique showed a significant decrease in PE species, which was compensated by increased levels of PC and PA [50, 52–54]. These mutant cells were also defective in GPI-anchor biosynthesis, failed to grow, were morphologically altered, and had impaired mitochondrial structure and function, demonstrating the importance of PE in parasite’s physiology [50]. Another particularity of *T. brucei* is that ether PE and diacyl–PE are produced by distinct enzymes, the ethanolamine-specific phosphotransferase *Tb*EPT and the choline/ethanolamine phosphotransferase *Tb*CEPT, respectively [55].

### *T. brucei* utilizes distinct pools of inositol for PI and GPI-anchor biosynthesis

PI biosynthesis in *T. brucei* can initiate from uptake of extracellular *myo*-inositol or from de novo produced inositol from glucose-6-phosphate [56, 57]. Notably, trypanosomes use distinct sources of *myo*-inositol for bulk PI and GPI-anchor production. Analysis of total cellular lipids of Golgi *myo*-inositol transporter *TbHMIT* mutant using the shotgun technique showed that *Tb*HMIT contributes to bulk PI production but not to GPI-anchor biosynthesis [58]. Conversely, *Tb*INO1, which converts glucose-6-phosphate into *myo*-inositol, provides the polar head for PI biosynthesis towards preferentially GPI-anchor biosynthesis rather than for bulk PI [57]. Inositol is subsequently condensed to DAG to form PI, which occurs via the action of the PI synthase *Tb*PIS localized in two organelles, the endoplasmic reticulum, where GPI-anchor biosynthesis occurs, and the Golgi apparatus, where bulk PI are produced [59]. *Tb*PIS is an essential gene for bloodstream trypanosomes. In vivo labelling of the *TbPIS* conditional knockout cells followed by quantitative and qualitative analysis of its whole lipidome by GLC–MS showed a significant decrease (70%) in all species of PI and a reduction in GPI-anchor levels as PI serves as a precursor for GPI-anchor biosynthesis.

### Defining the function of other glycerophospholipid metabolism enzymes by comparative lipidomics

Lipidomics was widely applied to address the role of lipid biosynthetic enzymes in glycerophospholipid metabolism. For example, *L. major* alkyl dihydroxyacetonephosphate synthase *LmADS* and dihydroxyacetonephosphate acyltransferase *Lm*DAT were found to be essential for the production of all ether glycerolipids as assessed by untargeted lipidomics, respectively [60, 61]. Untargeted lipidomics analysis using the shotgun method was employed to identify the substrate specificity of *L. major* PE methyltransferases *Lmj*PEM1 and *Lmj*PEM2 following heterologous expression in yeast lacking their respective, endogenous enzymes [49]. These analyses established that *Lmj*PEM1 added the first and second methyl group to PE, while *Lmj*PEM2 catalyzed all three methylation steps, although the addition of the first methyl group occurred very inefficiently. *T. brucei* possesses two initial acyltransferases, the glycerol-3-phosphate acyltransferase *Tb*GAT, which is dispensable for glycerolipid biosynthesis and growth of procyclic forms, and the dihydroxyacetonephosphate acyltransferase *Tb*DAT, which is essential for ether lipid production [62, 63]. The role of *T. brucei Tb*PLA_1_ in PC metabolism was investigated by global lipidomics investigation [64]. The *Tb*PLA_1_ null mutant was viable, and procyclic and bloodstream forms of the parasite were deficient in lysoPC synthesis. These studies established that *Tb*PLA_1_ enzyme functions in vivo in lysoPC production, containing mainly long-chain, polyunsaturated fatty acids.

## Mitochondrial fatty acid synthase II system is essential to *T. brucei*

*Trypanosoma brucei* has the ability to scavenge free fatty acids as well as to synthesize them using a mitochondrial type II fatty acid synthase for octanoate (a lipoic acid precursor) and longer fatty acids such as palmitate, and a microsomal elongase system (reviewed in [65]). In procyclic trypanosomes, RNAi depletion of the mitochondrial acyl carrier protein, a key component of the fatty acid synthesis complex, significantly reduced cytochrome-mediated respiration by inhibiting complexes II, III and IV, but not complex I of the electron transport chain [66]. A change in mitochondrial membrane composition may explain the altered mitochondrial morphology and membrane potential in the mutant. In fact, GC–MS analyses revealed a decrease in total cellular and mitochondrial PI, and mitochondrial PE quantities. The authors concluded that the mitochondrial fatty acid synthase system produces fatty acids needed for the generation of organellar glycerophospholipids, which are necessary for the activity of the electron transport chain and for the preservation of mitochondrial morphology and function.

## Biosynthesis of TAG in *T. brucei* is stimulated by exogenous fatty acid oleate

It has been proposed that TAG is important for the development of procyclic trypanosomes in the tsetse fly [67]. Thus, targeted lipidomics analysis was carried out to qualitatively identify the TAG species in *T. brucei* and their regulation in the presence or absence of oleate [67]. Oleate was found to stimulate the biosynthesis of the storage lipid TAG.

## *T. cruzi* scavenges host cholesterol and synthesizes cholesterol esters in reservosomes

*Trypanosoma cruzi* lacks the ability to synthesize cholesterol but produces instead ergosterol (reviewed in [11]). Thus, epimastigotes usurped cholesterol from the host and stored large quantities in form of cholesterol esters in dedicated lipid inclusions called reservosomes as revealed by targeted lipidomics by GC–MS analysis of a reservosome rich fraction [2]. Cholesterol esters were synthesized by the parasite itself and served as energy source for parasite differentiation. Upon exogenous lipid starvation, reservosomes’ free cholesterol was consumed, which was compensated by a rise in ergosterol biosynthesis. This study illustrates the importance of host cholesterol in *T. cruzi* development.

## Lipidomics and mechanism of drug action

### Miltefosine affects PL biosynthesis in *Leishmania*

Lipidomics approaches have been instrumental in elucidating the mechanism of action of anti-microbial drugs. Several lipid based drugs were shown to inhibit the growth of trypanosomatids [68]. Miltefosine, a choline analog, which was originally used as an anti-cancer drug, has recently been proven to be effective against leishmaniasis in clinical trials (reviewed in [6]). However, its mechanism of action is unclear. Based on its structure, it is predicted to affect lipid metabolism. Global lipidomics using LC–MS technology helped to establish the lipid profiles of miltefosine treated and resistant *L. donovani* strains. Short exposure to miltefosine lead to an overall induction in PL biosynthesis, particularly in PI, PE, and lysoPC (likely due to phospholipase A2 activation; [69–71]). However, miltefosine treatment lowered PC amounts [71, 72]. Surprisingly, drug resistant clones failed to manifest such drastic changes in PL profiles, demonstrating that the molecular basis of miltefosine resistance lays in distinct biological processes not related to lipid biosynthesis. Indeed, miltefosine resistant clinical isolates were defective in miltefosine uptake and carry mutation in the *LiMT* gene, which encodes a putative plasma membrane transporter [73].

### Antimony disturbs fatty acid synthesis

Pentavalent antimonials were the first line of anti-leishmanial drugs and drug resistance is quite common (reviewed in [74, 75]). The consequence of antimony exposure of *L. donovani* on lipid content lead to an increase in very long fatty acids and ergosterol levels based on GC–MS evaluation [76]. Fatty acid profiles of antimony resistant *L. chagasi* and *L. amazonensis* isolates showed that monosaturated C18:1Δ9c were increased in sensitive isolates while fatty acid 20:4Δ5,8,11,14 showed the opposite trend [77]. The authors proposed that these two fatty acids can be used as diagnostic markers for antimony resistance. Based on global lipidomics using LC–MS, antimonial resistance was also associated with altered lipid metabolism, suggesting that membrane composition of drug resistant parasites is extensively modified [78]. While the total lipid content was unchanged in both drug resistant and sensitive strains, unsaturated diacyl–PC and diacyl–PE levels were increased in drug resistant clones compared to those of sensitive ones, suggesting that desaturases may be induced by the drug. Lastly, antimony resistant clones produced minute amounts of sphingolipids, indicating that sphingolipid biosynthesis is affected in these parasites.

### OXPA blocks sphingolipid lipid biosynthesis

Global lipidomics was carried out by LC–MS to decipher the mechanism of action of 3-(oxalo[4,5-*b*]pyridine-2-yl)anolide (OXPA), a potent, novel anti-trypanosomal compound, which was optimized by structure–activity relationship investigation of a lead compound, previously identified from a drug library screening [79]. OXPA lead to the accumulation of ceramides, establishing that OXPA affects primarily sphingolipid metabolism [80]. Antibiotic myriocin, a serine palmitoyltransferase inhibitor, blocked cytokinesis and significantly decreased IPC biosynthesis in *L. brasiliensis* based on global lipidomics analysis [81]. From these studies, it can be deduced that sphingolipid metabolism offers a reasonable target for chemotherapeutic intervention.

### Sterol metabolism as target for chemotherapeutic compounds

Trypanosomatids are unable to synthesize cholesterol but instead produce ergosterol (reviewed in [11]). Imipramine, a widely used anti-depressant, exhibits anti-microbial effect against both cell stages, promastigotes and amastigotes, and inhibits sterol biosynthesis based on GC–MS analysis [82]. The effect of a natural chalcone, 2′6′-dihydroxymethoxylated chalcone, on lipid metabolism of *L. amazonensis* was investigated by global lipidomics analysis by GC–MS [83]. Exposure to this drug lead to accumulation of sterol precursors as well as to a reduction of C-14 demethylated and C-24 alkylated sterols, and decreased uptake of exogenous cholesterol.

Sterol analogs are potent anti-parasitic drugs that kill *T. brucei* and *T. cruzi* [84–86]. The compound 26-fluorolanosterol (26FL) inhibited ergosterol biosynthesis by blocking the target enzyme sterol C24-methyltransferase as per GC–MS analysis of their neutral lipids’ fraction [84]. Ketoconazole, 20 piperidin-2-yl-5α-pregnan-3β-20-*R*-diol (22,26-azasterol), and 24-(*R*,*S*),25-epiminolanosterol are FDA approved pharmaceuticals that are typically used against fungi. Ketoconazole inhibits the C24alpha demethylase, while 22,26-azasterol and 24-(*R*,*S*),25-epiminolanosterol block the δ24(25)—sterol methyl transferase of *T. cruzi* amastigotes. GC–MS analyses of total cellular lipids revealed that amastigotes contain primarily cholesterol (up to 80% of total cellular sterols) as well as 24-methyl-cholesta-7-en-3β-ol (ergosta-7-3β-ol) and its 24-ethyl analog, and smaller amounts of the precursor ergosta-7,24(28)dien-3β-ol [85]. Treatment of amastigotes with 22,26-azasterol or 24-(*R*,*S*),25-epiminolanosterol caused accumulation of C27 sterols. Ketoconazole drastically depleted the cellular content of 4-desmethyl sterol and concomitantly increased the 24-methyl-dihydrolanosterol and 24-methylen-dihydrolanosterol levels. Bisphosphonate risedronate (Ris), which is a drug currently used for the treatment of bone resorption disorder, displayed broader anti-parasitic activity [87, 88]. This compound specifically inhibits the farnesyl pyrophosphate synthase and thus, affects poly-isoprenoid metabolism [87]. GLC–MS analysis of *T. cruzi*’s lipidome revealed that the drug lessened endogenous sterols levels [86]. In addition, Ris manifested various pleiotropic effects such as mitochondrium swelling, disorganization of reservosomes and of the kinetoplast, vacuolization of the cytosol, premature induction of autophagy, and prevention of amastigote to trypomastigote differentiation. All together, these results support the idea that ergosterol metabolism is a suitable target for chemotherapeutic intervention in trypanosomatids.

## Lipidomics of *T. cruzi*’s host interaction

More recently, increased attention has been given to the role of lipids in pathogen–host interaction as well as in modulation of the host immune response by pathogens. In higher eukaryotes, lipid bodies, also termed lipid droplets or lipidic inclusions, are formed in response to host–pathogen interaction during the infection process and are the site of arachidonic acid’s conversion into inflammatory eicosanoids (reviewed in [89–91]). Lipid bodies are bounded by a single leaflet of phospholipids and are assembled in *T. cruzi* trypomastigotes after both host interaction and exogenous arachidonic acid stimulation. Shotgun lipidomics of lipid bodies of *T. cruzi* trypomastigotes revealed increased arachidonic acid quantities in these subcellular structures upon arachidonic acid stimulation [92]. Arachidonic acid-stimulated trypomastigotes released high amounts of prostaglandin E2, which results from arachidonic acid breakdown and acts as a potent immune modulatory lipid mediator that inhibits many aspects of innate and adaptive immunity. These studies established lipid bodies as key players in pathogen survival, virulence, inflammation, and interaction with the host.

Thromboxane A2 is the most effective vasoconstrictor as well as a proinflammatory agent that induces cytokine production by monocytes [93]. *T. cruzi*‘s pathological symptoms of the heart and blood vessels are similar to those caused by thromboxane A2 thus, the role of this eicosanoid in parasite’s pathogenesis was investigated [94]. Total lipid analysis by LC–MS of epimastigotes and of the blood of infected mice revealed the presence of thromboxane A2 and B2, the latter being the hydrolytic product of thromboxane A2. Thromboxane A2 is the predominant eiconasoid produced and released by all life stages of *T. cruzi* and accounts for up to 90% of the blood level in infected wild-type mice [94]. This study established that parasite’s thromboxane A2 affects the host immune response and determines the disease’s outcome.

## Conclusions

Lipidomics approaches have broadened our knowledge of the lipidome of trypanosomatids, which can be used as a reference for transgenic lines as well as biomarkers for detection of infection in diagnosis. This technique has enabled the identification of parasite’s specific lipid species and lipid biosynthetic pathways. However, the sensitivity of MS instrumentation can be improved to identify and quantify minute amounts of rare lipid species. Additionally, structure determination of stereoisomers and of unknown lipids still remain a challenge. Researchers in the field of trypanosomatids would also benefit from a parasite specific lipid database, such as LIPID MAPS for mammalian cells, which is missing. While the development of lipidomics has helped to shed light on the mechanism of action of several anti-parasitic drugs, the roles of parasite’s lipids in modulating the host’s immune system is still in its infancy. Only a few studies have be carried out with *T. cruzi* [92–94]; nothing is known about how *T. brucei* and *Leishmania* species influence the host’s lipidome, which can open some novel avenues for chemotherapeutic interventions. Altogether, the current knowledge resulting from lipidomics’ studies have so far led to the discovery of several kinetoplastid’s specific essential enzymes or metabolic pathways which can be exploited for pharmacological applications. The next phase consists in the identification or design of effective anti-microbial compounds, which specifically inhibit essential, parasites specific enzymes without negatively interfering with the host’s physiology. Several drug libraries are readily available for screening which will allow the selection of lead compounds. Alternatively, the input of computational biology in predicting or modelling of essential enzyme’s structure can facilitate the design of pharmacological drugs. These converging efforts will aid in the eradication of the debilitating diseases caused by trypanosomatids.

## Abbreviations

CFA: cyclopropane fatty acid
CID: collision-induced dissociation
CL: cardiolipin
EPC: ethanolaminephosphoceramide
ESI: electrospray ionization
FTICR-MS: Fourier-transform ion cyclotron resonance
GC: gas chromatography
GPI: glycosylphosphatidylinositol
IPC: inositolphosphoceramide
LC: liquid chromatography
MALDI-TOF: matrix assisted laser desorption/ionization coupled to time-of-flight
MS: mass spectrometry
OXPA: 3-(oxalo[4,5-*b*]pyridine-2-yl)anolide
PA: phosphatidic acid
PC: phosphatidylcholine
PE: phosphatidylethanolamine
PG: phosphatidylglycerol
PI: phosphatidylinositol
PL: phospholipid
PS: phosphatidylserine
Ris: bisphosphonate risedronate
SM: sphingomyelin
TAG: triacylglycerol
VSG: variant surface glycoprotein
